# Is primary aldosteronism a potential risk factor for aortic dissection? A case report and literature review

**DOI:** 10.1186/s12902-020-00601-9

**Published:** 2020-07-31

**Authors:** Ying Zhang, Fang Luo, Peng Fan, Xu Meng, Kunqi Yang, Xianliang Zhou

**Affiliations:** Department of Cardiology, Fuwai Hospital, National Center for Cardiovascular Diseases, Chinese Academy of Medical Sciences and Peking Union Medical College, No. 167, Beilishi Road, Beijing, 100037 China

**Keywords:** Aortic dissection, Case report, Hypertension, Primary aldosteronism

## Abstract

**Background:**

Primary aldosteronism (PA) increases the risk of cardiovascular morbidity, including stroke, coronary artery disease, atrial fibrillation, and heart failure. The relationship between primary aldosteronism and aortic dissection has rarely been reported. We report a case of aortic dissection caused by secondary hypertension from PA and review similar cases in the literature.

**Case presentation:**

A 56-year-old woman with a history of surgery for aortic dissection presented for follow-up of hypertension and a left adrenal mass. She had been diagnosed with hypertension and hypokalemia in 2003. Blood pressure had been controlled by antihypertensive medications. In 2009, she presented with chest and back pain; she was diagnosed with aortic dissection by computed tomography (CT). She underwent placement of an endovascular aortic stent graft. CT at that time showed a left adrenal mass with a diameter of 1 cm. In 2017, CT reexamination revealed that the left adrenal mass had grown to 3 cm in diameter. Laboratory data showed blood potassium 2.4 mmol/L (reference range: 3.5–5.3 mmol/L). The plasma aldosterone/renin ratio was elevated because of suppressed plasma renin and elevated serum aldosterone levels. Plasma aldosterone levels were not suppressed after taking captopril. Positron emission tomography/CT showed that the left adrenal tumor radiographic uptake was slightly increased (maximum standardized uptake value of 2.2), and metastasis was not detected. Laparoscopic adrenalectomy was performed, and an adrenocortical adenoma was confirmed histopathologically. After surgery, blood pressure and laboratory findings were within their reference ranges without any pharmacological treatment.

**Conclusions:**

Our patient and the literature suggest that PA is a potential cause of aortic dissection. Diagnosing PA in the early stages of the disease and early treatment are important because affected patients may be at increased risk of aortic dissection.

## Background

Primary aldosteronism (PA) is a group of disorders in which aldosterone production is inappropriately high [1]. Once thought to be rare, PA is now reported to be the most common cause of secondary hypertension, with a prevalence of 5 to 10% among patients with hypertension and up to 17 to 23% among patients with resistant hypertension [2, 3]. PA is commonly caused by an adrenal adenoma, unilateral or bilateral adrenal hyperplasia, or (rarely) adrenal carcinoma or inherited familial hyperaldosteronism [2]. Compared with primary hypertension, PA causes more end-organ damage and is associated with excess cardiovascular morbidity, including heart failure, stroke, myocardial infarction, and atrial fibrillation [4, 5]. PA also increases the risk of diabetes, metabolic syndrome, arterial wall stiffness, and left ventricular hypertrophy [4, 6, 7]. The cardiovascular system is affected even in normotensive individuals with early and mild PA [8]. Death resulting from cardiovascular causes is more common among patients with PA compared with matched control patients with primary hypertension [9]. Targeting PA treatment is effective in controlling blood pressure, protecting target organs, and improving cardiovascular outcomes [10].

Animal studies have demonstrated that aldosterone has a destructive effect on the aorta [11]. Recent clinical studies have also revealed that the diameter of the aorta in patients with PA is larger than that in patients with essential hypertension [12]. However, there are few studies on the relationship between PA and aortic dissection (AD), partly because of the low incidence of AD (35 cases per 100,000 people per year) and underdiagnosis of PA (screening rate: one case per 550 people) [13]. We report a new case of AD associated with secondary hypertension because of primary aldosteronism. In addition, similar cases in the literature are briefly summarized and discussed.

## Case presentation

**Fig. 1:**
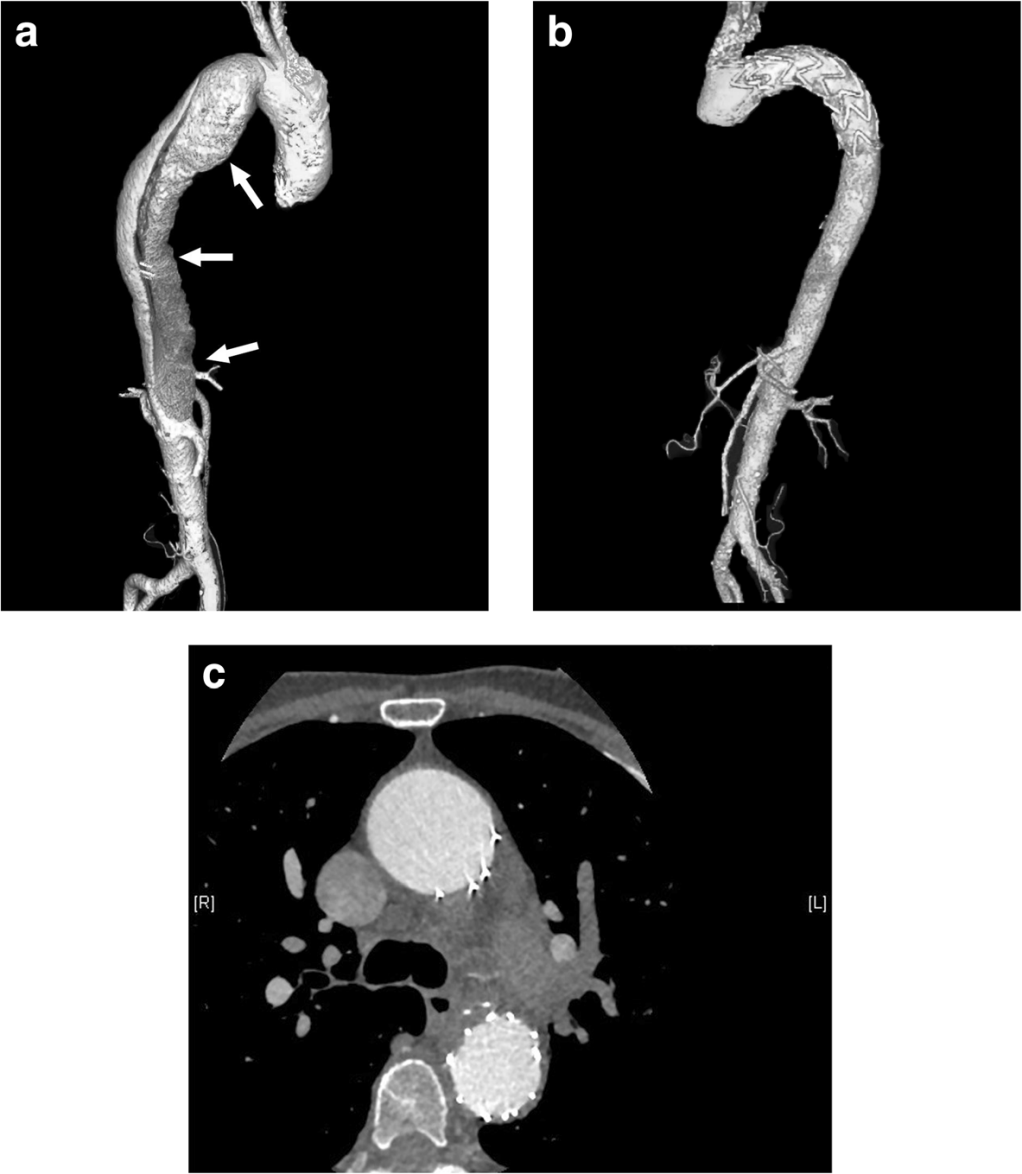
CT angiograms. **a** Dissection of the descending aorta in 2009 (arrows). **b**, **c** Repeat study in 2017 revealed no new dissection after TEVAR. CT: computed tomography; TEVAR: thoracic endovascular aortic repair

**Fig. 2:**
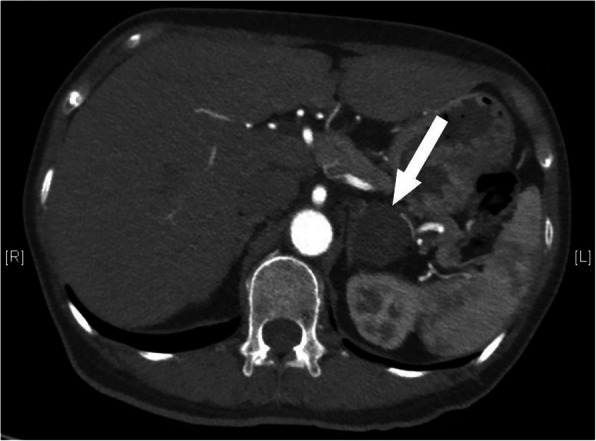
Abdominal CT. Left adrenal mass in 2017 (arrows). CT: computed tomography

A 56-year-old woman presented to our hospital with a history of a left adrenal mass and high blood pressure. She had been diagnosed with hypertension in 2003. Her blood pressure was controlled with amlodipine and was less than 140/90 mmHg at a dose of 5 mg daily. She reported frequent fatigue. She was admitted to the hospital, diagnosed with hypokalemia and treated with potassium supplements. In 2009, she presented with chest pain and back pain. A computed tomography (CT) angiogram demonstrated dissection of the descending aorta (Fig. 1a). Thoracic endovascular aortic repair (TEVAR) with a stent graft was successful. At the same time, she was found to have a left adrenal mass approximately 1 cm in diameter. She was started on valsartan to reduce blood pressure. Her blood pressure was 130–140/90–100 mmHg initially but gradually became difficult to control. Despite treatment with three antihypertensive drugs, her blood pressure still reached 180/110 mmHg. In 2017, repeat aortic CT revealed no new dissection (Fig. 1b, c). CT of the abdomen showed that the left adrenal mass had grown to more than 3 cm in diameter (Fig. 2).

She was investigated further in our hospital. Monitoring of 24-h ambulatory blood pressure demonstrated an average blood pressure of 182/102 mmHg. Laboratory data showed blood potassium 2.4 mmol/L (3.5–5.3 mmol/L). The circadian rhythm of cortisol, overnight 1 mg dexamethasone suppression test, sex hormone levels, as well as catecholamines and metabolites were normal (Table 1). Abdominal CT showed no abnormality in the kidney and renal arteries. The diagnosis of primary hyperaldosteronism was confirmed by a captopril challenge test (Table 2). Plasma aldosterone/renin ratio was elevated due to a suppressed plasma renin and elevated serum aldosterone level. Plasma aldosterone levels were not suppressed after taking captopril. Positron emission tomography (PET)/CT showed that the left adrenal tumor radiographic uptake was slightly increased, with a maximum standardized uptake value (SUVmax) of 2.2, and no metastases were detected.

**Table 1:** Laboratory findings on admission

Parameters	Values	Reference ranges
Serum potassium (mmol/L)	2.4	3.5–5.3
Creatinine (umol/L)	98.7	44.0–133.0
Testosterone (ng/dL)	11.69	14–76
Progesterone (ng/mL)	0.08	< 0.73
Estradiol (pg/mL)	20.3	< 32.2
Prolactin (ng/mL)	4.73	1.8–20.3
Luteotropic hormone (mIU/mL)	33.41	15.9–54.6
Follicle-stimulating hormone (uIU/mL)	62.37	23.0–116.3
Plasma norepinephrine (ng/mL)	0.43	0.104–0.548
Plasma epinephrine (ng/mL)	0.005	0.02–0.08
Plasma dopamine (ng/mL)	0.005	< 0.03
Urinary Normetanephrine (μg/24 h)	394	< 1464
Urinary Metanephrine (μg/24 h)	71	< 394
Plasma total cortisol (μg/dL) 8 am-16 pm-0 am	14.5–7.8-7.2	4.3–22.4 (7-9 am) 3.1–16.7 (3-5 pm)
ACTH (pg/mL) 8 am-16 pm-0 am	9.7- < 5- < 5	< 46
1 mg DST (ug/dL) 8 am basal day-8 am post DST	7.6–5.8	

*ACTH* adrenocorticotropic hormone, *DST* dexamethasone suppression test

**Table 2:** Results of the ARR tests, posture stimulation test and captopril challenge test

	PAC (ng/dL)	DRC (mU/L)	ARR (ng/dl: mU/L)
Supine position	150.0	0.9	166.7
Upright position	175.0	2.9	60.3
Before captopril challenge	61.1	1.8	33.9
After captopril challenge	60.4	5.4	11.2

*PAC* plasma aldosterone concentration, *DRC* direct renin concentration, *ARR* aldosterone-to-renin ratio

**Fig. 3:**
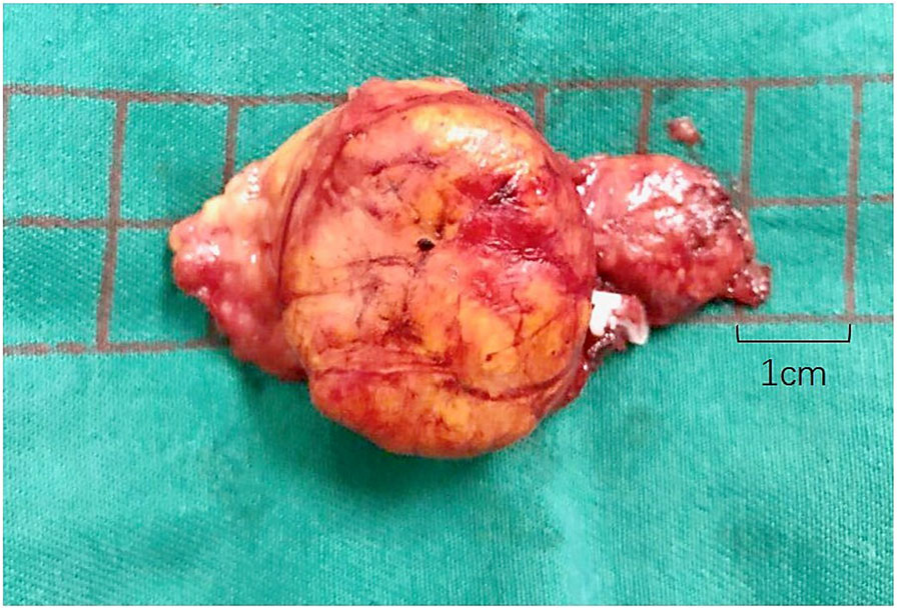
Left adrenal gland after laparoscopic adrenalectomy in 2017. The solid nodule had a diameter of 3 cm and was confirmed histopathologically to be an adrenocortical adenoma

After discussing the medical and surgical management options, the patient decided to initially opt for medical management. She took spironolactone and two other antihypertensive drugs. Five days later, the patient’s blood pressure fell to 142/85 mmHg, and potassium had risen to 3.93 mmol/L. Despite initial good control of hypertension, blood pressures rose again and after 8 m of medical treatment, the patient elected to undergo left laparoscopic adrenalectomy. The tumor, which was about 3 cm in diameter, was in the middle of the adrenal gland (Fig. 3). Adrenocortical adenoma was confirmed histopathologically.

The postoperative course was uneventful, and the patient’s blood pressure and serum potassium remained normal without medication.

## Discussion and conclusions

In this article, we present an incidental finding of AD in a patient with PA. The patient was known to have hypertension and hypokalemia for many years but was not screened for PA during this time. Although the hypertension was controlled (less than 140/90 mmHg) by taking antihypertensive drugs, dissection still occurred. We consider that PA is a risk factor for AD independent of hypertension. Because of the high mortality and poor prognosis of AD, the association of PA with AD deserves greater attention.

An electronic literature search in PubMed was performed to identify case reports relating to PA with AD. Search words included “primary hyperaldosteronism” and “aortic dissection”. All references of included reports and relevant reviews were screened manually for additional potential eligible cases. The results were limited to full-text articles published in English. Seven available reports were included in the review [14–20] (Table 3). The PA features of these patients are summarized as follows: In the PA subtype classification, five patients had adrenal adenoma, one patient had nodular cortical hyperplasia, and one patient had glucocorticoid-remediable aldosteronism. In patients with adrenal adenoma, the diameter of the nodule ranged from 1.0 to 3.0 cm, and the majority were left-sided (six out of seven, 85.7%). AD commonly occurs in the sixth and seventh decades of life [21]. However, in the patients with PA, the mean age of onset of AD was 37.8 ± 12.0 years (range: 10–48 years). The relatively young age suggests that the aldosterone hypersecretion possibly contributed to AD. In these cases, PA patients with AD often had a delay in diagnosis and treatment. Two patients were diagnosed with PA following AD, and three were diagnosed with PA with AD simultaneously. Only three patients had PA diagnosed before AD, and in all the treatment of PA was inadequate. According to epidemiological studies, normokalemic hypertension constitutes the most common presentation of PA, with only a minority of patients with PA (9–37%) being hypokalemic and hypokalemia probably occurring in only the more severe cases [2]. In our summary case reports, all cases in which plasma or serum potassium values were available had hypokalemia. It therefore appears likely that patients with AD tend to have more severe forms of PA. The above evidence raises the possibility that the damage of aldosterone to blood vessels and leading to AD may be independent of blood pressure; furthermore, excessive aldosterone may be a risk factor for AD.

**Table 3:** The summary of the reported cases of aortic dissection in patients with primary aldosteronism

Case	Study	Age/Sex	Age of diagnostic			Serum potassium	AD type	PA			PA Treatment before AD
			HTN	PA	AD	(mmol/l)	(D/S)	Type	Side	Size (cm)	
1	Shimizu et al., 1983 [13]	37/F	30	34	37	2.7	I/A	Adrenal adenoma	Right	1.5	Yes, spironolactone for 1 year
2	Lam et al., 1999 [14]	39/M	39	39	43	2.7	I/A	Nodular cortical hyperplasia	Left	1.4	No
3	Safi et al., 1999 [15]	39/F	15	39	39	2.5–3.0	II/A	Adrenal adenoma	Left	1	No
4	Ahmed et al., 2007 [16]	48/M	–	48	48	3.2	III/B	–	Left	2	Yes, aldosterone antagonist for 1 month
5	Harvey et al., 2010 [17]	39/M	29	39	39	2.5	III/B	Adrenal adenoma	Left	–	Yes, aldosterone antagonist for 6 months
6	Hirai et al., 2010 [18]	38/M	–	38	38	1.9	III/B	Adrenal adenoma	Left	1	No
7	Shahrrava et al., 2016 [19]	24/M	10	18	10	–	–	Glucocorticoid remediable aldosteronism	–	–	No
8	Our case	56/F	42	56	48	2.4	III/B	Adrenal adenoma	Left	3	No

*HTN* hypertension, *PA* primary aldosteronism, *AD* aortic dissection, *D/S* Debakey/Stanford

The actions of aldosterone in the vasculature have been studied over the last few years, and the mechanisms are beginning to be clarified. Animal studies show the importance of aldosterone in promoting vascular inflammation, resulting in endothelial dysfunction [22–24]. In addition, aldosterone recruitment of vascular inflammatory cells leads to the development of atherosclerosis [25]. Inflammation also increases the density of fibronectin and collagen in the media of arteries, resulting in arterial stiffness [26]. Aldosterone leads to medial smooth muscle cell proliferation and vascular remodeling, which is enhanced by endothelial injury [27]. The increase in the fibronectin/elastin ratio reflects alterations in extracellular matrix content [22], leading to increased medial thickness and media-to-lumen ratio, which is detected not only in rat aortas but also in the resistance arteries of patients with PA [26, 28, 29].

Aldosterone exerts its effect on blood vessels by activating the mineralocorticoid receptor (MR) within the vascular endothelium and vascular smooth muscle. Rickard et al. [30] demonstrated that endothelial function was impaired by the presence of MR in endothelial cells in a mouse model, indicating that endothelial MR played an important role in aldosterone-induced endothelial dysfunction. In another study, a wire-induced carotid injury model using wild-type mice and mice with an inducible smooth muscle cell (SMC)-specific deletion of the MR. The results show that SMC-MR is necessary for aldosterone-induced vascular remodeling independent of effects on blood pressure. SMC-MR contributes to the induction of SMC vascular endothelial growth factor receptor 1 in the area of vascular injury and contributes to aldosterone-enhanced vascular placental growth factor expression [31].

MR antagonists, such as spironolactone and eplerenone, have a beneficial effect through inhibition of the vascular MR [11]. Spironolactone improves the structure and increases tone in the cerebral vasculature of spontaneously hypertensive stroke-prone rats [32]. In addition, spironolactone increases the middle cerebral artery lumen diameter and reduces the wall/lumen ratio in spontaneously hypertensive stroke-prone rats compared [33]. MR antagonists also act on the aldosterone/MR pathway in promoting vascular changes involved in atherogenesis. Eplerenone improves endothelial function and reduces superoxide generation in diet-induced atherosclerosis [34]. Eplerenone also decreases lesion size in early atherosclerosis in apolipoprotein E–deficient mice; targeting aldosterone by blocking its receptor has anti-atherosclerotic effects [35].

Our case and data from the literature indicate that PA is a possible risk factor for AD independent of hypertension. This case underlines the importance of early detection of PA in the prevention of severe cardiovascular complications such as AD, especially in high-risk groups of hypertensive patients and those with hypokalemia. Furthermore, the possible association between AD and PA should be elucidated in future studies.

## Data Availability

All data generated or analyzed during this study are included in this published article.

## Abbreviations

ACTH: Adrenocorticotropic hormone
AD: Aortic dissection
ARR: Aldosterone-to-renin ratio
CT: Computed tomography
DST: Dexamethasone suppression test
DRC: Direct renin concentration
D/S: Debakey/Stanford
HTN: Hypertension
MR: Mineralocorticoid receptor
PA: Primary aldosteronism
PAC: Plasma aldosterone concentration
PET: Positron emission tomography
SMC: Smooth muscle cell
SUVmax: Maximum standardized uptake value
TEVAR: Thoracic endovascular aortic repair
